# Spindle Cell Hemangioendothelioma of the Temporal Muscle Resected with Zygomatic Osteotomy: A Case Report of an Unusual Intramuscular Lesion Mimicking Sarcoma

**DOI:** 10.1155/2011/481654

**Published:** 2011-09-06

**Authors:** Tomohiro Minagawa, Takeshi Yamao, Ryuta Shioya

**Affiliations:** ^1^Department of Plastic and Reconstructive Surgery, Asahikawa Kosei General Hospital, Hokkaido, Asahikawa 078-8211, Japan; ^2^Department of Plastic and Reconstructive Surgery, Hokkaido University Graduate School of Medicine, Hokkaido, Sapporo 060-0815, Japan

## Abstract

Spindle cell hemangioendothelioma (SCH) was originally described by Weiss and Enzinger (1986) as a low-grade angiosarcoma resembling both cavernous hemangioma and Kaposi's sarcoma. Recent studies suggest that SCH is a benign neoplasm or reactive lesion accompanying a congenital or acquired vascular malformation. Most SCHs present as one or more nodules affecting the dermis or subcutis of the distal extremities. Few reports describe SCH of the head and neck region; even fewer note intramuscular SCH. Here, we describe a case of SCH involving the temporal muscle mimicking soft tissue sarcoma, who had a successful surgical treatment with a coronal approach and zygomatic osteotomy.

## 1. Introduction

Spindle cell hemangioendothelioma (SCH) was first described in 1986 by Weiss and Enzinger as a vascular neoplasm, characterized by cavernous blood vessels and spindled areas reminiscent of Kaposi's sarcoma [1]. SCH typically presents as a single tumor or multiple nodules involving the dermis and subcutaneous tissues of the distal extremities [2, 3]. The head and neck region is rarely involved; only six cases have been previously reported [4]. Intramuscular SCH is considered extremely rare [5]. We present a case of SCH arising in the temporal muscle that resembles a malignant soft tissue tumor.

## 2. Case Presentation

**Figure 1:**
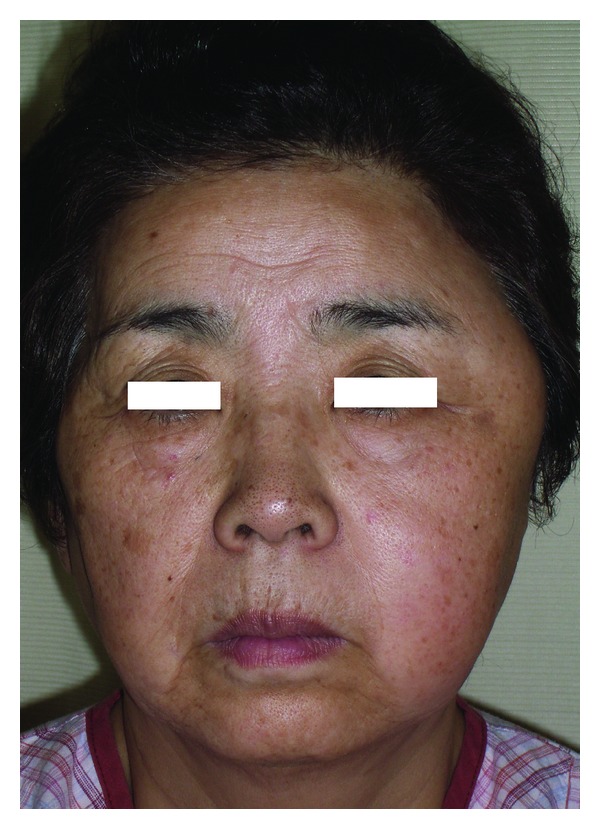
Preoperative view of the patient.

 A 67-year-old female complained about swelling of the left temporal area with a 4-month history. She had no previous history of trauma in the region. On examination, there was a firm, hemispherical mass 40 mm in diameter in the temporal area; overlying skin appeared normal. The mass was immobile against underlying bone. Trismus or facial palsy was not observed (Figure 1).

**Figure 2:**
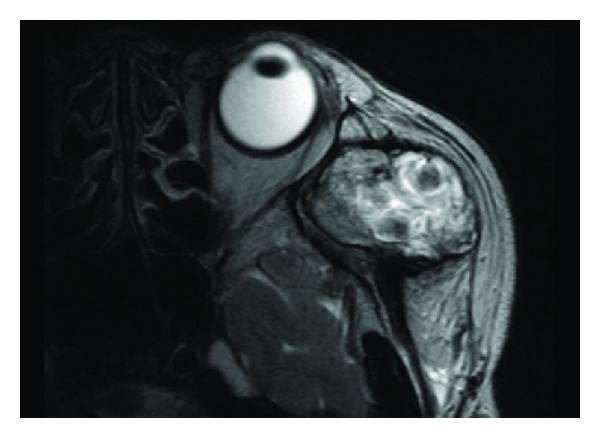
Preoperative MRI on T2-weighted image showing high signal intensity admixed with irregular low signal areas in left temporal fossa.

**Figure 3:**
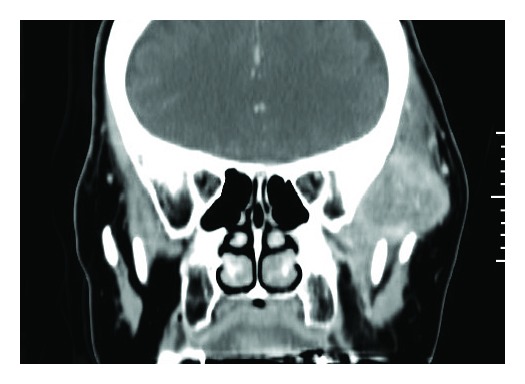
Preoperative contrast CT showing ill-demarcated low-density mass with enhancement of the adjacent temporal muscle.

Magnetic resonance imaging (MRI) revealed a relatively well-demarcated mass in the left temporal muscle, which showed low signal intensity on T1-weighted images and high signal intensity admixed with irregular low signal areas on T2-weighted images (Figure 2). Computed tomography showed an ill-demarcated round mass. Heterogeneous enhancement of the mass and adjacent temporal muscle was observed between the coronoid process and temporal fossa (Figure 3). A highly vascularized soft tissue tumor with malignant potential was suspected based on the aggressive features indicated by radiology and rapid growing of the lesion.

An incisional biopsy was performed under general anesthesia to evaluate histology. The specimen revealed proliferation of dilated small vessels with hemorrhage associated with inflammatory components. Abnormal mitotic figures were not observed. Histological features suggested a benign or intermediate vascular tumor, particularly angiolymphoid hyperplasia with eosinophilia.

**Figure 4:**
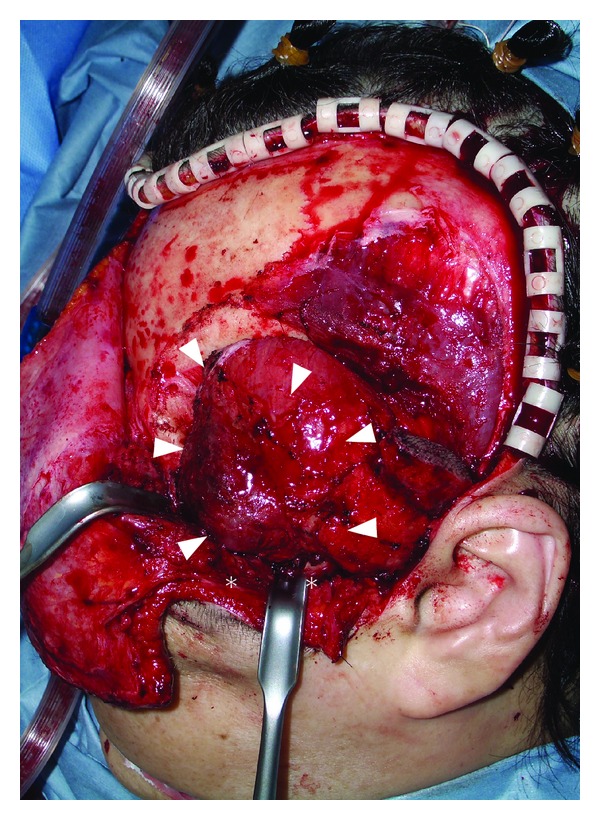
Intraoperative view demonstrating intramuscular tumor (arrows) with the assistance of zygomatic osteotomy (asterisks).

**Figure 5:**
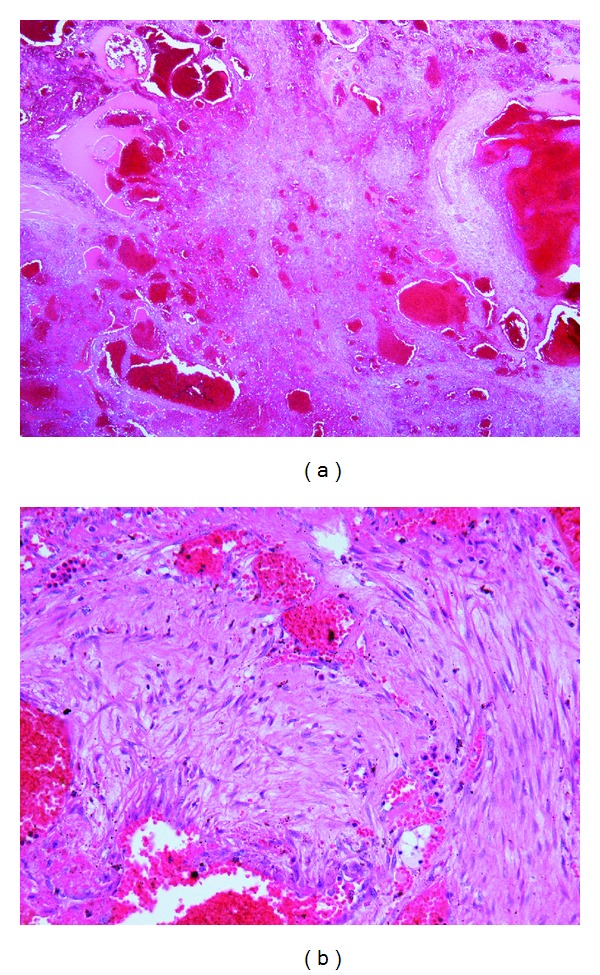
Microscopic findings of the spindle cell hemangioendothelioma. (a) Low-power view of the lesion showing cavernous blood vessels filled partly or completely with erythrocytes or thrombus admixed with cellular zones (hematoxylin-eosin, original magnification ×40). (b) High-power view of juxtaposition of the cavernous and cellular areas illustrating blood-filled dilated vessels lined by flattened endothelial cells and spindle-shaped cellular components. There is no evidence of abnormal mitotic activity or nuclear atypia in spindled cells (hematoxylin-eosin, original magnification ×100).

**Figure 6:**
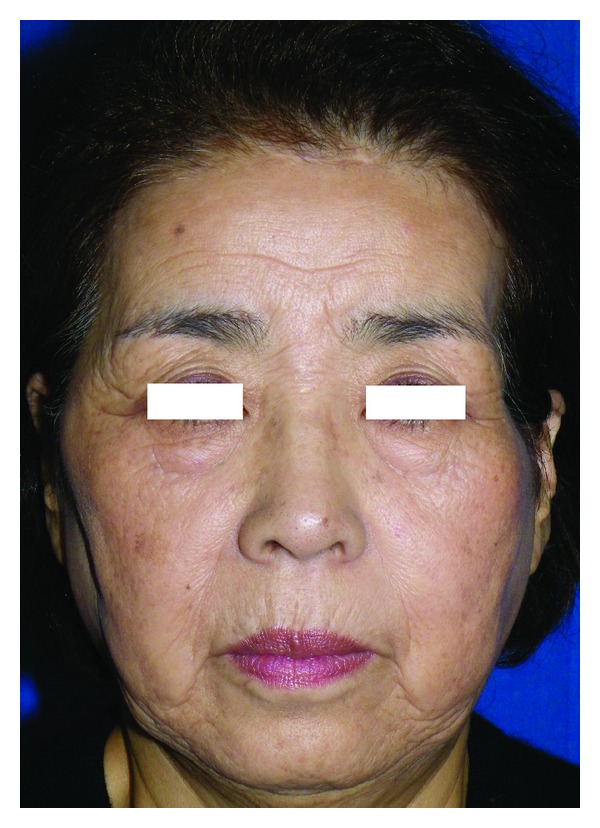
Postoperative view at 24 months followup.

Surgical excision was performed under general anesthesia. First, a hemicoronal incision of the scalp was made; subsequently, the superficial surface of the temporal muscle and zygomatic arch were exposed. A solid nodule was palpated in the temporal muscle and continued beneath the zygomatic arch (Figure 4). Osteotomy of the zygomatic arch was performed, and the bone fragment was temporarily removed. The entire temporal muscle was bluntly dissected from adjacent bones, revealing no bony involvement. Then, the temporal muscle was severed just distal to the coronoid process and at the distal insertion, removing the tumor with the muscle en bloc. Finally, the zygomatic arch fragment was replaced and fixed with 3/0 absorbable sutures. A suction drain was inserted in the temporal fossa, and the scalp wound was closed in two layers. The surgical specimen showed thin-walled cavernous vessels lined by endothelial cells, hemorrhage, and spindle-shaped cells with inflammatory cell infiltration. Abnormal mitotic figures or cellular atypia was not observed (Figures 5(a) and 5(b)). These histological characteristics led to a diagnosis of SCH. Postoperative course was uneventful. No signs of local recurrence were observed during two years of followup. Postoperative complications such as facial palsy, trismus, or alopecia have not been observed. The patient was satisfied with the aesthetic result, though a mild depression was observed in the temporal area (Figure 6).

## 3. Discussion

This case of SCH involving temporal muscle is extremely rare. According to the 78 series of SCH reported by Perkins and Weiss, four cases occurred in the head and neck region, while 62 cases involved extremities [6]. To date, six cases of SCH of the head and neck have been reported [4]. Because most SCHs present as nodules involving dermis and subcutaneous tissue [2, 3], few reports exist on intramuscular SCH [5]. Recent studies classify SCH as a reactive lesion that arises in association with a local congenital or acquired vascular malformation, rather than a neoplastic lesion [7–9]. In the present case, pathogenesis of temporal muscle involvement remains unclear given that angiography was not performed preoperatively. However, contrast CT revealed high vascularity of the entire temporal muscle, which may indicate that the SCH was associated with vascular abnormalities in the muscle.

 Local recurrence is prevalent in SCH. Of the reports by Perkins and Weiss, 58 percent of patients developed recurrences in the same general area of the body [6]. Because of such high rate of recurrence, SCH was originally considered a low-grade angiosarcoma [1]. However, recent evidence indicates that the majority of lesions are partially or exclusively intravascular [6]. Therefore, “recurrence” is defined as contiguous or multifocal spreading of the lesion along an affected vessel [6, 7, 10]. Overall, prognosis is excellent and no metastases from SCH have been reported to date [6]. However, considering the location of the SCH occupying the temporal and infratemporal fossa in the present case and since SCH can be associated with vascular abnormalities, marginal excision carried the risk of local “recurrence” and massive blood loss. Thus, we performed en bloc excision of the entire affected muscle [5] with a coronal approach and zygomatic osteotomy. The intra- and postoperative course was uneventful, with satisfactory aesthetic results and no evidence of recurrence. Further followup may be required.
